# A novel method for causal structure discovery from EHR data and its application to type-2 diabetes mellitus

**DOI:** 10.1038/s41598-021-99990-7

**Published:** 2021-10-25

**Authors:** Xinpeng Shen, Sisi Ma, Prashanthi Vemuri, M. Regina Castro, Pedro J. Caraballo, Gyorgy J. Simon

**Affiliations:** 1 Institute for Health Informatics, University of Minnesota, Minneapolis, MN USA; 2 Department of Medicine, University of Minnesota, Minneapolis, MN USA; 3 Department of Radiology, Mayo Clinic, Rochester, MN USA; 4 Division of Endocrinology, Mayo Clinic, Rochester, MN USA; 5 Department of Internal Medicine, and Department of Health Sciences Research, Mayo Clinic, Rochester, MN USA

**Keywords:** Risk factors, Computational models, Machine learning, Statistical methods

## Abstract

Modern AI-based clinical decision support models owe their success in part to the very large number of predictors they use. Safe and robust decision support, especially for intervention planning, requires causal, not associative, relationships. Traditional methods of causal discovery, clinical trials and extracting biochemical pathways, are resource intensive and may not scale up to the number and complexity of relationships sufficient for precision treatment planning. Computational causal structure discovery (CSD) from electronic health records (EHR) data can represent a solution, however, current CSD methods fall short on EHR data. This paper presents a CSD method tailored to the EHR data. The application of the proposed methodology was demonstrated on type-2 diabetes mellitus. A large EHR dataset from Mayo Clinic was used as development cohort, and another large dataset from an independent health system, M Health Fairview, as external validation cohort. The proposed method achieved very high recall (.95) and substantially higher precision than the general-purpose methods (.84 versus .29, and .55). The causal relationships extracted from the development and external validation cohorts had a high (81%) overlap. Due to the adaptations to EHR data, the proposed method is more suitable for use in clinical decision support than the general-purpose methods.

## Introduction

Diagnostic tools based on artificial intelligence (AI) have recently demonstrated human-like performance^1–4^, owing their high performance to their ability to synthesize information from many features. Consistent with this observation, national initiatives such as the Precision Medicine Initiative^5^ and the Learning Health Systems^6^ encourage the inclusion of a wide-range of information about the patient into the decision making process. Increasingly, clinical decision support systems start to include treatment planning and selection tools^7^. Such tools require causal knowledge, not merely the associations (correlations). Intervening on correlates rather than causal factors of the disease leads to lack of efficacy, under- or overtreatment, and in worst case, to iatrogenic harm^8^.

The gold standard for discovering causal relationships is conducting a randomized clinical trial or elucidating the underlying biochemical pathways. In many cases, clinical trials are impractical, unethical, if not outright impossible. Computational causal structure discovery (CSD) methods to discover causal relationships have demonstrated great success in many domains^9–11^ and their application to EHR data could offer a solution for causal discovery from observational real world medical data. However, to unlock their full potential, these general-purpose algorithms need to be adapted to address study design and data quality challenges specific to the EHR data.

We propose an algorithm with three adaptations. First, we incorporate *study design considerations.* EHR data as it exists in the system does not follow any study design. Billing codes in particular are recorded for reimbursement purposes and do not distinguish between new incidences and pre-existing conditions. Understanding this difference is critical for study design. Second, *time stamps can be unreliable.* The time stamp of a diagnosis often does **not** coincide with the onset time of the disease, but rather reflects the documentation time. In some cases, the temporal ordering of diseases may be reversed. Partly for this reason, general purpose CSD algorithms applied to the EHR data occasionally report “causal” relationships that are in the opposite direction of the natural disease progression. Third, *general-purpose CSD methods sometimes fail to orient edges.* Even when a clear causal direction exists and is not masked by data artifacts, CSD algorithms can have difficulty distinguishing the cause from the effect due to statistical equivalence^12^. Leveraging the longitudinal nature of EHR data and incorporating time information as part of the causal discovery process can enhance the identification of edge orientation.

In this paper, (1) we propose a data transformation procedure that distinguishes new incidences from pre-existing conditions, which allows us to determine the temporal order of the disease-related events despite the inaccurate (or rather noisy) timestamps in the EHR data. (2) We then present modifications to an existing CSD method, (Fast) Greedy Equivalence Search (GES)^13,14^, to infer the direction of causal relationships more robustly using longitudinal information and takes the above study design considerations into account.

We demonstrate this methodology through the clinical example of type-2 diabetes mellitus (T2D), its risk factors and complications. T2D is an exceptionally well-studied disease with numerous clinical trials having produced a vast knowledge base, making the evaluation of the methodology possible. The goal of this work is not to uncover new causal relationships in diabetes, but to present a novel methodology for discovering causal relationships from EHR data that are sufficiently robust to support model development for clinical decision support tools. While we use T2D as our use case, we expect our methods to generalize to other diseases, typically chronic diseases, that exhibit similar characteristics and suffer from the same EHR shortcomings.

## Methods

### Study source and population

**Figure 1:**
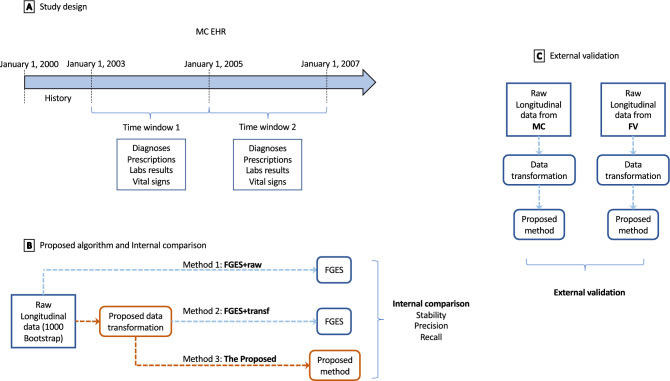
Study design and evaluations. (**A**) Overview of the study design for Mayo Clinic (MC) EHR. (**B**) The workflow of the internal evaluation. Three methods FGES + raw, FGES + transf, and the proposed algorithm were compared using stability, precision, and recall. Orange color highlights the proposed method (Method 3). (**C**) The workflow of external comparison. The proposed method was applied to two datasets, MC and M Health Fairview (FV), and the resulting graphs were compared.

This retrospective cohort study utilized EHR data sets from two independent health systems, Mayo Clinic (MC) in Rochester, Minnesota and M Health Fairview (FV) in Minneapolis, Minnesota. Two 2-year time windows 2003–2004 and 2006–2007 for MC; and 2008–2009, and 2011–2012 for FV were defined. Dates for the time windows differed between MC and FV due to data availability. We extracted diagnoses, prescriptions, laboratory results, and vital signs from the two EHR data sets with the same inclusion and exclusion criteria: patients must have at least two blood pressure measurements, one before the first time window and one after the second time window; aged 18 + at the end of the first time window; and sex and age must be known. Figure 1A shows an overview of the study design of MC EHR (the study design for FV is similar). We used the MC EHR as the development cohort.

### Variables

Diagnosis codes are aggregated into the disease categories of obesity, hyperlipidemia, pre-diabetes, type 2 diabetes mellitus, coronary artery disease, myocardial infarction, heart failure, chronic renal failure, cerebrovascular disease, and stroke based on ICD-9 and codes following our previous work^15^. Medications indicated for the above conditions were rolled up into NDF-RT therapeutic subclasses. Relevant laboratory results and vital signs were categorized based on cutoffs from the American Diabetes Association guidelines^16^.

### Causal structure discovery

A relationship between two events is *causal* if manipulating the earlier event causes the other (later) event to change. For example, prescribing a medication reduces the probability of downstream events (complications). Causation differs from association. For example, blood sugar is associated with risk of stroke: diabetic patients with higher blood sugar have a higher risk of stroke; however, this relationship is likely not causal in diabetic patients since attempts to reduce the risk of stroke by reducing blood sugar consistently failed in clinical trials^17,18^. If two events share a common cause (a *confounder*) and are not otherwise causally related, then manipulating one event will not affect the other variable as long as the common cause remains unchanged. The confounder can be observed or latent. The term **causal structure** refers to the set of all existing causal relationships among all events and can be visualized as a graph. The causal graph consists of nodes, which corresponds to events, and the nodes are connected by edges that denote causal relationships. General-purpose CSD methods are designed to work with observational data to derive a causal structure that are consistent with the joint probability of the data.

Several general-purpose CSD algorithms have been proposed and the interested reader is referred to the Supplements II where we present an overview of the major methods. In this work, we focus on (Fast) Greedy Equivalence Search (FGES) as the comparison method, because we previously found it to outperform other CSD methods^19^. Briefly, FGES finds the optimal causal graph by a greedy search guided by a goodness-of-fit score (e.g. BIC or BDeu) over all possible graphs. Particularly, it starts with an empty graph, and iteratively adds individual edges that maximize the score given the current graph, until adding edges no longer improves the score. Then, FGES iteratively removes individual edges that maximizes the score, until edge removal ceases to improve the score. The output of FGES is a pattern, which can contain undirected edges, where the causal effect direction could not be determined due to statistical equivalency. FGES has good mathematical properties and been shown to be consistent under a set of assumptions^14,20^.

### Proposed methods

The workflow of the proposed methods is described in Fig. 1B, method 3 (colored in orange). We propose two methods, a data transformation and a causal search method. The former method transforms the longitudinal EHR data into **disease-related events**, so that we can determine the temporal ordering of events (diseases) despite inaccuracies in the EHR data and extracts all pairs of diseases where a clear precedence ordering exists. The search method constructs the causal graph using the transformed data and the set of precedence pairs.

### Data transformation method

A disease-related *event* is defined as a diagnosis, a prescription, an abnormal lab result, or abnormal vital sign. An event is *incident* if it occurs in the second time window but is not present in the first time window although the patient is observed in the first time window. Conversely, a disease event is *pre-existing* if the patient presented with it in or before the first time window. An event *A precedes* another event *B* if among patients who have both *A* and *B* in the second time window, *B* is significantly more likely to be incident than *A*. Note that precedence implies neither causation nor association; however, if a causal effect exists, it must follow the precedence direction. Formal mathematical definitions of these concepts can be found in the Supplement I. The output from this step is (i) an event-based data set consisting of the incident and pre-existing conditions for each patient in each of the two time windows, (ii) a set ${\mathcal{C}}$ of precedence relationships of all pairs $\left( {v_{i} ,{ }v_{j} } \right)$ of events for which event $v_{i}$ clearly precedes $v_{j}$.

### The proposed CSD search Algorithm

Given ${\mathcal{C}}$, we construct the causal graph ${\mathcal{G}}$ by iteratively adding edge $\left( {v_{i} ,{ }v_{j} } \right){ }$ from ${\mathcal{C}}$ that maximizes the goodness of fit of ${\mathcal{G}}$. The orientation of this edge must be consistent with the precedence relationship, namely from $v_{i}$ to $v_{j}$. The goodness of fit is defined by the BIC criteria. Let $X^{\left( 1 \right)} ,{ }X^{\left( 2 \right)}$ denote the data sets collected in the two distinct time windows, where $X^{\left( 2 \right)}$ follows $X^{\left( 1 \right)}$. The likelihood of the ${\mathcal{G}}$ is

1 $$\begin{aligned} {\mathcal{L}}\left( {{\mathcal{G}}|X^{{\left( 1 \right)}} ,~X^{{\left( 2 \right)}} } \right) & = \text{P}\left( {~X^{{\left( 2 \right)}} ,~X^{{\left( 1 \right)}} |{\mathcal{G}}} \right) = \text{P}\left( {~X^{{\left( 2 \right)}} ~|~X^{{\left( 1 \right)}} ,{\mathcal{G}}} \right)~\text{P}\left( {~X^{{\left( 1 \right)}} |{\mathcal{G}}} \right) \\ & = ~\mathop \prod \limits_{s} \mathop \prod \limits_{{v~ \in {\mathcal{V}}}} \text{P}\left( {v_{s}^{{\left( 2 \right)}} |x_{s}^{{\left( 1 \right)}} ,~{\mathcal{G}}} \right)\text{P}\left( {~x_{s}^{{\left( 1 \right)}} |{\mathcal{G}}} \right)\begin{array}{*{20}c} { = ~\mathop \prod \limits_{s} \mathop \prod \limits_{{v~ \in {\mathcal{V}}}} \text{P}\left( {v_{s}^{{\left( 2 \right)}} |~pa\left( {v,~{\mathcal{G}}} \right)_{s}^{{\left( 1 \right)}} } \right)\text{P}\left( {~x_{s}^{{\left( 1 \right)}} |{\mathcal{G}}} \right),} \\ \end{array} \\ \end{aligned}$$

where $x_{s}^{\left( t \right)}$ is the observation vector for subject s at the cross-section *t*; $v_{s}^{\left( t \right)}$ is the observation of variable (event) *v* for subject *s* at the cross-section *t*; and $pa\left( {v, {\mathcal{G}}} \right)_{s}^{\left( 1 \right)}$ is the observation vector for the parents of *v* in the causal structure ${\mathcal{G}}$, at cross Sect. 1 for subject *s*.

The algorithm estimates $P\left( {v_{s}^{\left( 2 \right)} |pa\left( {v, {\mathcal{G}}} \right)_{s}^{\left( 1 \right)} } \right)$ using logistic regression on the subjects that do not have *v* at the first cross section and are under observations for both cross sections. For subjects who have *v* at the first cross section, the probability of having *v* at the second cross section is 1. Since G represents the transition graph, the term $P\left( { x_{s}^{\left( 1 \right)} |{\mathcal{G}}} \right)$ is a constant.

Finally, the BIC score is

2 $$\begin{array}{*{20}c} {BIC\left( {\mathcal{G}} \right) = - 2lnL\left( {{\mathcal{G}}|X^{\left( 1 \right)} ,X^{\left( 2 \right)} } \right) + ln\left( {\text{n}} \right)\left| {\mathcal{G}} \right|,} \\ \end{array}$$

where n is the number of observations that are common in the two cross sections, and $\left| {\mathcal{G}} \right|$ is the number of edges in the causal structure ${\mathcal{G}}$.

Algorithm 1 describes the proposed algorithm for constructing the causal graph ${\mathcal{G}}$. ${\mathcal{G}}$ is a directed acyclic graph (DAG), with nodes representing variables and edges representing causal effects between a pre-existing and an incident variable.
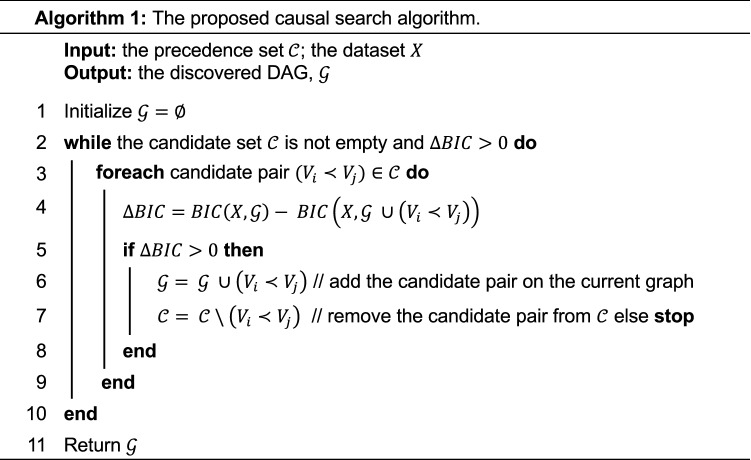


### Statement of human rights and informed consent

The study was approved by both Mayo Clinic and University of Minnesota Institutional Review Board (IRB). Informed consent was obtained from all patients. All relevant guidelines and regulations were followed.

## Evaluation

### Clinical evidence

The standard way to evaluate CSD methods is to compare the resulting graph to a gold standard graph. However, such a gold standard graph does not exist and possibly many relationships are unknown. However, there exists (1) Associative Evidence: a large body of observational studies documenting risk factors and outcomes for diabetes. Results from these studies have already been distilled into summaries^21^. (2) Clinical trials can support both the existence (positive) and also the lack (negative) of hypothesized causal relationships. We compiled a list of causal relationships from clinical trials considering 175 clinical trials with a primary endpoint of any of the conditions we studied, including composite end points. We excluded trials with inclusion criteria that are too strict (trial results would not generalize to our population) and the interventions that are out of the scope of our study. 14 trials remained yielding 19 positive and 18 negative causal relationships. These trials and the evidence they produced are listed in Supplement III, Table S1. These relationships are used as causal evidence to compute recall.

### Internal evaluation

We evaluated the method and the resulting graphs from the following four perspectives.

#### Stability

We run 1000 bootstrap replicas on the development cohort. An edge has ambiguous orientation if it is present in at least half of the 1000 graphs (edge is not noise) and both orientations appear in at least 30% of the graphs that contain this edge (it does not have a dominant direction). We report the percentage of ambiguous edges.

#### Precision

Based on the causal graph derived from the training cohort, an edge is **incorrect** if there is no associative evidence of a relationship between the two events; or if causal evidence specifically indicates the lack of a causal relationship. We define *precision* as one minus the proportion of incorrect edges among the discovered edges.

#### Causal recall

Causal recall is computed on a single graph discovered from the training cohort, quantifying the percentage of the known causal relationships discovered. A known causal relationship from *A* to *B* is discovered if there is a node in the graph that maps to *A*, another node that maps to *B* and (a) a direct causal relationship *A* → *B* in the graph exists or (b) a causal path *A* → *X* → *B* exists and no causal evidence states that in patients with *X*, *A* does not cause *B*. For example, if the evidence states that blood pressure (without specifying whether it is systolic or diastolic) increases the risk of stroke, then the path sbp → cevd → stroke would satisfy this relationship.

#### Associative recall

Associative recall is also computed on a single graph discovered from the training cohort and it quantifies the percentage of known associative relationships that can be explained by the discovered causal graph. An associative relationship between A and B is explained by the graph if there is a node in the graph that maps to *A*, another node that maps to *B*, and a path between *A* and *B* exists in the graph*.*

### External validation

We performed 1000 bootstrap replications on both data sets independently using the proposed method. On each data set, all edges from the 1000 graphs were pooled, resulting in two sets of pooled edges. We compared these two sets and pointed out the edges that were discordant between the MC and FV data, as shown in Fig. 1C.

### Method comparison

Figure 1B depicts an overview of the method comparison. Three methods are compared, (1) *FGES* + *raw* FGES is applied directly to the raw data; (2) *FGES* + *transf* data is transformed using the proposed transformation method and FGES is applied to the transformed data; and (3) *Proposed* the proposed search algorithm is applied to the transformed data. Comparing FGES + raw and FGES + transf isolates the effect of the proposed transformation method, and comparing FGES + transf and Proposed highlights the effect of the proposed search algorithm.

## Results

### Baseline characteristics

Table 1 presents descriptive statistics for the MC and FV data sets at the end of the first time window and incidence rates for the diseases in the second window. Differences between datasets are tested through the t-test (for age) and the chi-square test (all other variables).

**Table 1:** Characteristics of the MC and FV data sets.

	MC (N = 57,332)		FV (N = 79,486)		P-value
	Events in window 1	New events in window 2	Events in window 1	New events in window 2	
**Demographics**					
Age	48.1 (18.2)		50.4 (14.6)		0.000
Male	0.43		0.34		0.000
Ethnicity white	0.92		0.93		0.000
**Vitals and labs**					
BMI ≥ 25 and < 30	27.1	2.9	27.5	3.4	0.097
BMI ≥ 30	32.6	3.6	43.1	4.9	0.000
SBP ≥ 140	10.3	3.4	4.5	2.9	0.000
DBP ≥ 90	2.3	1.0	1.6	1.2	0.000
LDL ≥ 130	18.4	3.6	15.4	4.3	0.000
HDL abnormal	20.2	1.7	24.6	3.0	0.000
Trigl ≥ 150	22.6	3.7	17.6	4.3	0.000
FPG ≥ 100 and < 125	24.4	7.2			
FPG ≥ 125	11.9	3.7			
A1c ≥ 5.7 and A1c < 6.5			6.8	0.6	
A1c ≥ 6.5			7.0	0.9	
**Diagnoses**					
Hypertension (HTN)	28.4	5.6	30.6	8.4	0.000
Obesity (Ob)	11.5	1.2	11.3	1.3	0.320
Hyperlipidemia (HL)	31.9	8.3	36.4	9.4	0.000
Pre-diabetes mellitus (predm)	0.9	3.5	2.4	2.4	0.000
Diabetes mellitus (DM)	7.9	5.1	9.5	4.3	0.000
Chronic renal failure (CRF)	1.2	0.2	0.2	0.3	0.000
Congestive heart failure (CHF)	2.4	1.7	1.2	1.4	0.000
Coronary artery disease (CAD)	9.4	3.5	5.6	3.4	0.000
Myocardial infarction (MI)	2.4	1.2	0.9	1.6	0.000
Cerebrovascular disease (CeVD)	3.6	2.3	1.8	1.4	0.000
Stroke	1.2	1.1	0.6	1.0	0.000
**Treatments**					
Hypertension	20.6	8.3	31.5	13.9	0.000
Hyperlipidemia	15.7	8.0	24.6	9.1	0.000
Diabetes mellitus	4.4	2.4	7.2	4.3	0.000

For age, mean (sd) is indicated; for the disease-related events, percentage (%) of positive is indicated. New events rate at the second time windows is reported.

BMI: Body mass index; SBP: systolic blood pressure; DBP: diastolic blood pressure, Trigl: triglycerides, FPG: fasting plasma glucose; A1c: hemoglobin A_1c_.

### Directional stability

The proposed data transformation reduced the percentage of ambiguously oriented edges from 45 to 24%, and finally, the proposed search method eliminated ambiguously oriented edges (Table 2).

**Table 2:** Directional stability.

Method	Number of distinct edges	Ambiguously oriented (%)
FGES + raw	125	45
FGES + transf	75	24
Proposed	64	0

The table shows the number of distinct edges that appeared in half of the 1000 bootstrap replications, and the percentage of ambiguously oriented edges.

### Correctness and completeness

**Figure 2:**
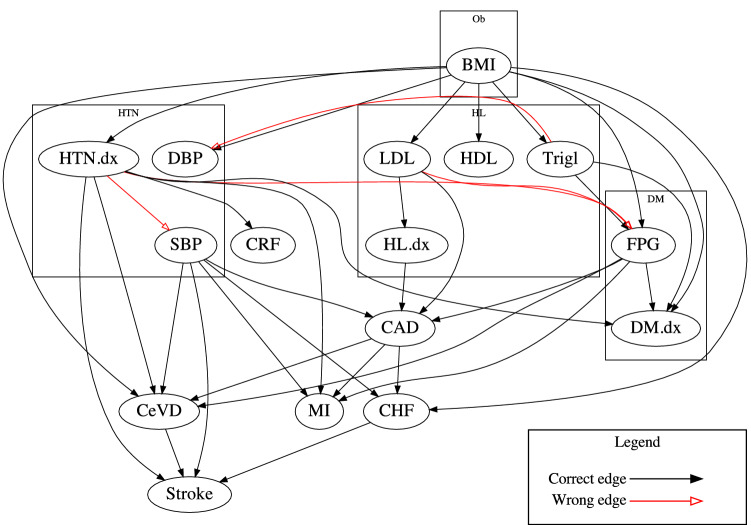
Causal graph discovered by the proposed method. The ‘.dx’ suffix indicates diagnosis of the disease. The abbreviations of the diseases and lab results can be found in Table 1.

Table 3 shows the precision, associative recall and causal recall of the graphs discovered by the three methods. All three methods achieved almost perfect recall; FGES + raw achieved the lowest precision of 0.294: less than third of the events reported as causally related are even associated. By using the proposed transformation, the precision increased to 0.55, but almost half of the reported causal relationships are still incorrect. Finally, the proposed method achieved a precision of 0.838. We present the causal graph discovered by the proposed methods in the Fig. 2. Incorrect edges are colored in red.

**Table 3:** Metrics from clinical evidence.

	Precision	Associative recall	Causal recall
1. FGES + raw	0.294	1.000	1.000
2. FGES + transf	0.549	0.985	1.000
3. Proposed	0.838	1.000	0.947

### External validation

We compared the graphs discovered from the MC and FV data sets. There are 74 distinct edges that were discovered from at least one of the data sets. Sixty (81%) edges coincided across the two datasets, while 14 (19%) differed. Table 4 shows the discordant edges, the percentage of bootstrap iterations in which the edge was present and the main reason for the discordance.

**Table 4:** External validation.

Edge	Discovery %		Reason
	MC	FV	
HDL → Trigl	0	91.7	There is no clear precedence relationship, the two events often coincide
HTN.dx → CRF	88.5	0.1	
Trigl → DM.dx	100	0	
Trigl → HL.tx	100	0	
LDL → HL.dx	72.1	0	
FPG.125 → DM.dx	100	0	FV uses A1c, not FPG
Trigl → FPG.125	99.5	0.2	
DBP → HTN.tx	91.5	0	The criteria for diagnosis and treatment are institution specific
SBP → HL.tx	99.3	1.7	
SBP → HTN.tx	100	29.1	
Trigl → HTN.tx	83.7	0	
CHF → MI	0	67.6	SBP is a common cause for CHF and MI, but at FV, this effect was too weak in 68% of the bootstrap iterations
HL.dx → Trigl	0	87.6	While the main driver of Trigl is BMI, at FV, the diagnosis of HL helps explain the variation in Trigl
HL.tx → CAD	0	74.3	LDL drives both HL treatment and CAD

The ‘.tx’ suffix denotes the treatment, and ‘.dx’ suffix denotes the diagnosis of the disease. The abbreviations of the diseases and lab tests can be found in Table 1. The table describes the edges that were discordant between the Mayo Clinic (MC) and M Health Fairview (FV) data sets. It shows the percentage of the bootstrap iterations in which the edge was discovered at MC and FV and a brief reason for the discrepancy.

There are three broad reasons for differences in edges. The main reason, affecting half of the edges was that of policy differences. These include preferred lab results (A1C vs FPG) and decisions regarding therapeutic interventions. The second reason, affecting four edges, is a lack of clear precedence in the relationships among the events. For example, the abnormal Trigl → HL treatment edge was not discovered at FV because the first abnormal Trigl precedes or follows the HL treatment in statistically equal proportions. The final reason, affecting the remaining three edges, is differential degree of confounding between the two sites. For example, SBP is a confounder of CHF and MI. When the algorithm fails to detect the SBP → MI edge, the effect of SBP on MI was shown through CHF (which depends on SBP more than MI). For the HL diagnosis → Trigl edge, the common cause is BMI, and for the HL treatment → CAD edge, it is LDL. The reason for differential confounding was likely a combination of population and institutional differences as well as data artifacts.

## Discussion

We proposed a new data transformation method and a new search algorithm specifically designed for EHR data. We showed how the resulting graph achieved close to 90% precision (90% of the edges were correct), almost 100% recall (the graph could explain all known associations and almost all known causal relationships), and the graph was remarkably stable in face of data perturbation (no edge disappeared or changed direction). Due to its built-in facility, our method outperformed general purpose methods by a large margin.

While the two graphs from the two independent health systems are reassuringly similar, small differences exist. None of these differences implies an incorrect physiological or pathophysiological effect. Among the 14 edges that differed, seven captured differences between the population and the institutions, such as institution-specific triggers for prescriptions and the use of different laboratory tests for the same purpose (fasting plasma glucose versus A1c). Depending on the goal of the modeling, it may be desirable to include such differences. We believe that the discovered causal graphs offer adequate information about causal (including confounding) factors to support the development of clinical decision support models and can also support clinical research efforts.

The proposed algorithm achieved such high performance because it could compensate for errors in the EHR data and it incorporated study design considerations. Problems caused by incorrect time stamps and diseases appearing in the reverse order are alleviated by reducing the overall reliance on time stamps. The study design with its two-year windows allows for (even large) errors in the time stamp and once a disease is recognized as pre-existing by the data transformation method, its subsequent time stamps are irrelevant. Time stamps that appear in the reverse order tend to have a small gap (time to schedule and complete a diagnostic procedure), so they likely fall into the same two-year window. Study design considerations, namely that billing codes do not distinguish between incident and pre-existing conditions as well as whether a patient is under observation or not, are addressed through the data transformation method. The ability of the search algorithm to produce a DAG is achieved through using precedence relationships to orient edges that have equal probability in both orientations. Precedence relationships in turn rely on the pre-existing/incident status of the disease as determination by the data transformation method.

### Generalizability beyond diabetes

The proposed method was demonstrated on type 2 diabetes, but it can generalize to other applications as long as the target application benefits from some of the improvements: reducing the impact of inaccuracies in the EHR data, accounting for the temporal ordering of events and distinguishing pre-existing and incident conditions. The method assumes that pre-existing diseases persist during the second time window.

### Future work

The algorithm requires longitudinal data with at least two time windows. Different diseases and their symptoms might manifest at different rates, incorporating this knowledge into the discovery process may enhance the performance of the algorithms. Secondly, the proposed methods may be able to capture the effect of medication changes when a study design of multiple (more than two) time windows is applied. The current implementation assumes a single incidence of a disease, or that the diseases persists during the study period. Another possible extension could relax this assumption, allowing for transient conditions that can have multiple incidences in the study period. Thirdly, variable semantics (such as SBP and DBP being measures related to hypertension) is an essential component of the proposed algorithm, but it is not always available in a computable form. Further, both datasets in this study are from the Midwest with a predominantly white patient population. The generalizability of the discovered causal relations can be further tested by examining a broader patient population.

## Conclusions

We have demonstrated that the graph produced by the proposed transformation and search algorithm is more stable across bootstrap iterations and as complete as other methods yet it contained substantially fewer errors (had higher precision) than graphs produced by general-purpose methods. The resulting graph was successfully validated using longitudinal EHR data from an independent health system. We conclude that the proposed method is more suitable for use in clinical studies using EHR data.

## Supplementary Information

Supplementary Information.

## Data Availability

The data that support the findings of this study are not publicly available since they contain patient health information. Authorization to access patient data can be requested from the Mayo Clinic and University of Minnesota Institutional Review Board.
