# Supplementary material for: A novel method for causal structure discovery from EHR data and its application to type-2 diabetes mellitus
Source: Sci Rep. 2021 Oct 25;11:21025. doi: 10.1038/s41598-021-99990-7 (PMC8546093; doi:10.1038/s41598-021-99990-7)
Supplement: Supplementary file 1 — Supplementary Information. [file 41598_2021_99990_MOESM1_ESM.docx]

**A novel method for Causal Structure Discovery from EHR data and its application to type-2 diabetes mellitus**

Xinpeng Shen^a^, Sisi Ma^a,b^, Prashanthi Vemuri^c^, M. Regina Castro^d^, Pedro J. Caraballo^e^, Gyorgy J. Simon^a,b*^

^a^Institute for Health Informatics, University of Minnesota, Minneapolis, MN, USA

^b^Department of Medicine, University of Minnesota, Minneapolis, MN, USA

^c^Department of Radiology, Mayo Clinic, Rochester, MN, USA

^d^Division of Endocrinology, Mayo Clinic, Rochester, MN, USA

^e^Department of Internal Medicine, and Department of Health Sciences Research, Mayo Clinic, Rochester, MN, USA

**SUPPLEMENT**

We provide four pieces of supplementary information. The first one describes the details of the method, the second one provides a review of the existing causal discovery methods and the third section summarizes our clinical trial literature review. Finally, the fourth table contains the abbreviations used in the manuscript.

**Supplement I:**

**Data Transformation Method and mathematical definitions**

We first define the concepts of precedence and describe our method for determining the temporal ordering of two diseases. If two diseases have a causal relationship, the precedence relationship between them plays an important role in determining the causal effect direction: the causal effect must travel from the earlier to the later disease. As the first step, we extract all pairs of diseases from the data set with a clear precedence relationship. Next, we define a likelihood function and describe how the causal graph is constructed from the set of precedence relationships.

*Precedence*

Let $\mathcal{V}=\{V_{1}, V_{2},\ldots,V_{m}\}$ denote a set of $m$ variables (diseases or abnormalities). Consider patients who already developed both variables $V_{i}$ and $V_{j}$ in cross section 2. The proportion of patients who newly developed $V_{j}$ but had pre-existing $V_{i}$ is

$$\begin{aligned} P\left( {v_{i}}^{\left( 1 \right)}=1, {v_{j}}^{\left( 1 \right)}=0 | {v_{i}}^{\left( 2 \right)}=1, {v_{j}}^{\left( 2 \right)}=1 \right),\#\left( 1 \right) \end{aligned}$$

where the superscript represents the cross section. Conversely, the proportion of patients who newly developed $V_{i}$ after pre-existing $V_{j}$ among those who have both $V_{i}$ and $V_{j}$ in cross section 2

is

$$\begin{aligned} P\left( {v_{i}}^{\left( 1 \right)}=0, {v_{j}}^{\left( 1 \right)}=1 | {v_{i}}^{\left( 2 \right)}=1, {v_{j}}^{\left( 1 \right)}=1 \right).\#\left( 2 \right) \end{aligned}$$

For a pair of variables $V_{i}$ and $V_{j}$ in cross sections 1 and 2, $V_{i}\prec V_{j}$ (pron. “$V_{i}$ **precedes** $V_{j}$”) if and only if:

$$\begin{aligned} P\left( {v_{i}}^{\left( 1 \right)}=1, {v_{j}}^{\left( 1 \right)}=0 | {v_{i}}^{\left( 2 \right)}=1, {v_{j}}^{\left( 2 \right)}=1 \right)> \\ P\left( {v_{i}}^{\left( 1 \right)}=0, {v_{j}}^{\left( 1 \right)}=1 | {v_{i}}^{\left( 2 \right)}=1, {v_{j}}^{\left( 2 \right)}=1 \right)\#\left( 3 \right) \end{aligned}$$

Note that precedence implies neither causation nor association; however, if a causal effect exists, it must follow the precedence direction. This requirement follows from the assumption that causal effects do not progress backwards in time.

**Supplement II:**

**Overview of general-purpose CSD algorithms**

Existing algorithmic causal discovery methods typically fall into three categories: constraint-based (e.g. PC[1], FCI[2 3]), score-based (e.g. FGES), and hybrid methods (e.g. MMHC[4]). The constraint-based methods rely on conditional independence relationships to discover causal relationships. The PC algorithm is one of the most popular constraint-based CSD method. The PC algorithm starts with a fully connected undirected graph, and iteratively removes edges based on the results from an independence testing method. The search procedure concludes by orienting the remaining edges according to a set of rules. The FCI algorithm is an extension of the PC algorithm. Unlike PC, which assumes the absence of unobserved confounding, under certain circumstances for some edges, FCI has the ability to verify that indeed no latent confounding is present.

Graph Equivalence Search (GES) is a well-known score-based CSD method. Score-based methods aim to find an optimal causal graph by iteratively and greedily optimizing a goodness-of-fit score, which in case of the GES algorithm is the quasi-Bayesian information criterion (BIC). It optimizes this score by starting with an empty structure without any edges, and adding edges iteratively until adding further edges no longer improves the fit. Then, GES tries to remove edges one at a time as long as the fit improves. The output is a graphical representation of the causal structure, called a pattern[1]. Like a directed acyclic graph (DAG), a pattern consists of nodes and edges, but unlike a DAG, where every edge has a direction, edges in a pattern can be undirected. Edges are undirected when the scores corresponding to the different orientations of the edge are the same. From a different point of view, a pattern represents a set of DAGs satisfying the same independence criteria. Theoretically, GES has been shown to be consistent under a set of assumptions, including no unobserved confounders, and infinite sample size.

Hybrid methods are the combination of score-based and constraint-based methods. For instance, GFCI[5] method inherits GES’s searching procedure to search for the skeleton (no directed edge) and uses FCI’s orientation procedure to orient edges.

Recent CSD methods extend the previous methods by relaxing many of the assumptions: handling non-linear casual relationships[6], cyclic causal relationships[7], individualized causal structures[8 9]. Additionally, integration of deep learning into CSD[10-14] has opened new methodological directions including the use of generating adversarial networks (GANs)[15-17] and reinforcement learning (RF)[18-20]. Despite the advances in methods for causal structure discovery, to our knowledge, no method can overcome the specific challenges posed by the EHR data. Our proposed method is inspired by the score-based methods and designed to work effectively with EHR data.

**Supplement III**

| **Table S1.** Constructing the gold standard graph | | | |
| --- | --- | --- | --- |
| **Target** | **Risk factors** | **Associative edges** | **Evidence from RCT** |
| DM | BMI, HTN, FPG, Trigl | BMI, HTN, SBP, DBP, FPG, Trigl | SBP, DBP (HOPE[21]) |
| Cevd | BMI, HTN, HL, DM, CAD, CHF, MI | BMI, HTN, SBP, DBP, LDL, HL, FPG, DM, CAD, CHF, MI | Trigl (VA-HIT[22]\|HDL) |
| Stroke | BMI, HTN, HL, DM, CAD, CHF, MI, CEVD | BMI, HTN, SBP, DBP, LDL, HL, FPG, DM, CAD, CHF, MI, CeVD | SBP (SPS3-BP\|stroke, ACCORD BP[23]\|DM) !FPG (ACCORD[24]\|DM, UKPDS 33[25]\|DM) !Trigl (ACCORD LIPID[26]\|LDL,DM) !BMI (LOOK AHEAD[27]\|DM) LDL (HPS statin[28]) |
| CAD | HTN, HL, DM, BMI, Trigl | BMI, HTN, SBP, DBP, LDL, HL, FPG, DM, Trigl | Trigl (VA-HIT\|HDL,FIELD[29]\|DM) BMI (LOOK AHEAD\|DM) LDL (HPS statin) |
| MI | HTN, HL, Trigl, DM, BMI, CAD | BMI, HTN, SBP, DBP, LDL, HL, Trigl, FPG, DM, CAD | SBP, DBP (HOPE) !SBP,DBP (SPS3-BP\|stroke,ACCORD BP\|DM) FPG (ACCORD\|DM, UKPDS 33\|DM) !Trigl (ACCORD LIPID\|LDL,DM) Trigl (VA-HIT\|HDL,FIELD\|DM) !BMI (LOOK AHEAD\|DM) LDL (HPS statin) |
| CHF | CAD, MI, HTN, DM, BMI | BMI, HTN, SBP, DBP, CAD, MI, FPG, DM | SBP, DBP (HOPE) !FPG (ACCORD)\|DM, UKPDS 33\|DM, ADVANCE[30]\|DM) !SBP,DBP (ACCORD BP\|DM) !Trigl (ACCORD LIPID\|LDL,DM) !BMI (LOOK AHEAD\|DM) |
| CRF | DM, HTN, CHF | HTN, SBP, DBP, FPG, DM, CHF | FPG (ADVANCE\|DM, DCCT[31]\|T1D) !FPG (UKPDS 33\|DM) SBP,DBP (Benazepril in Severe CKD[32]\|!DM, RENAAL[33]\|DM) |

Table S1 summarizes the clinical evidence we collected. We use two types of evidence: associative and causal. Associative evidence was obtained from a collection of documents that summarizes current knowledge about diseases, its risk factors, treatments, and other aspects. This collection is procured by Mayo Clinic. The second type of evidence, causal evidence, is derived from clinical trials. We considered 175 clinical trials with a primary endpoint including any of the conditions we study, as well as composite endpoints. We excluded trials with inclusion criteria that are too strict (trial results would not generalize to our population) and the interventions that are out of the scope of our study. 14 trials remained yielding 19 positive and 18 negative relationships.

The first column in Table S1 is the name of the target disease. The second column shows the risk factors from Mayo Clinic patient care and health information page and the third column contains their mapping to our EHR-derived conditions. (e.g. HTN is mapped to HTN, SBP and DBP.) It has the form “(!)A([RCT name]|C)”, where an exclamation mark indicates the relationship does not exist, A is the risk factor, and C represents pre-existing conditions used as inclusion criteria for the RCT. For example, for the target disease CHF, the expression “!FPG (ACCORD)|DM”, suggests that FPG is not a risk factors of CHF in diabetic patients, because the ACCORD trial found that aggressive management of FPG failed to reduce the patients’ risk of CHF.

**Supplement IV**

**Table S2: Abbreviations used in the manuscript**

| ***Abbreviation*** | ***Definition*** |
| --- | --- |
| EHR | Electronic Health Record |
| FHS | Fairview Health Services |
| SD | Standard Deviation |
| AI | Artificial Intelligence |
| F(GES) | Fast (Greedy Equivalence Search) |
| T2D/DM | Type-2 Diabetes Mellitus |
| T1D | Type-1 Diabetes Mellitus |
| MC | Mayo Clinic |
| CSD | Causal Structure Discovery |
| BMI | Body Mass Index |
| SBP | Systolic Blood Pressure |
| DBP | Diastolic Blood Pressure |
| LDL | Low-density Lipoprotein Cholesterol |
| HDL | High Density Lipoprotein |
| Trigl | Triglyceride |
| FPG | Fasting Plasma Glucose |
| A1c | Glycated Hemoglobin |
| HTN | Hypertension |
| OB | Obesity |
| HL | Hyperlipidemia |
| CRF | Chronic Renal Failure |
| CHF | Congestive Heart Failure |
| MI | Myocardial Infarction |
| CeVD | Cerebrovascular Disease |

**Reference**

1. Spirtes P, Clark G, Scheines R. *Causation, Prediction, and Search*: The MIT Press, 2000.

2. Peter S, Christopher M, Thomas R. Causal inference in the presence of latent variables and selection bias. Proceedings of the Eleventh Conference on Uncertainty in Artificial Intelligence 1995:499–506

3. Colombo D, Maathuis MH, Kalisch M, Richardson TS. LEARNING HIGH-DIMENSIONAL DIRECTED ACYCLIC GRAPHS WITH LATENT AND SELECTION VARIABLES. The Annals of Statistics 2012;**40**(1):294-321

4. Tsamardinos I, Brown LE, Aliferis CF. The max-min hill-climbing Bayesian network structure learning algorithm. Machine Learning 2006;**65**(1):31-78 doi: 10.1007/s10994-006-6889-7.

5. Ogarrio JM, Spirtes P, Ramsey J. A Hybrid Causal Search Algorithm for Latent Variable Models. In: Alessandro A, Giorgio C, Cassio Polpo C, eds. Proceedings of the Eighth International Conference on Probabilistic Graphical Models. Proceedings of Machine Learning Research: PMLR, 2016:368--79.

6. Hoyer PO, Janzing D, M. MJ, Jonas P, Bernhard Sl. Nonlinear causal discovery with additive noise models. 2009:689--96

7. Sanchez-Romero R, Ramsey JD, Zhang K, Glymour MRK, Huang B, Glymour C. Estimating feedforward and feedback effective connections from fMRI time series: Assessments of statistical methods. Network neuroscience (Cambridge, Mass.) 2019;**3**(2):274-306 doi: 10.1162/netn_a_00061.

8. Jabbari F, Visweswaran S, Cooper GF. Instance-Specific Bayesian Network Structure Learning. In: Václav K, Milan S, eds. Proceedings of the Ninth International Conference on Probabilistic Graphical Models. Proceedings of Machine Learning Research: PMLR, 2018:169--80.

9. Jabbari F, Cooper G. An Instance-Specific Algorithm for Learning the Structure of Causal Bayesian Networks Containing Latent Variables, 2020:433-41.

10. Zheng X, Aragam B, Ravikumar PK, P. XE. DAGs with NO TEARS: Continuous Optimization for Structure Learning. 2018:9472--83

11. Yu Y, Chen J, Gao T, Yu M. DAG-GNN: DAG Structure Learning with Graph Neural Networks. In: Kamalika C, Ruslan S, eds. Proceedings of the 36th International Conference on Machine Learning. Proceedings of Machine Learning Research: PMLR, 2019:7154--63.

12. Zheng X, Dan C, Aragam B, Ravikumar P, Xing E. Learning Sparse Nonparametric DAGs. In: Silvia C, Roberto C, eds. Proceedings of the Twenty Third International Conference on Artificial Intelligence and Statistics. Proceedings of Machine Learning Research: PMLR, 2020:3414--25.

13. Lachapelle S, Brouillard P, Deleu T, Lacoste-Julien S. *Gradient-Based Neural DAG Learning*, 2019.

14. Young J, Andrews B, Cooper G, Lu X. *Learning Latent Causal Structures with a Redundant Input Neural Network*, 2020.

15. Bellot AavdSM. Conditional Independence Testing using Generative Adversarial Networks. 2019:2202--11

16. Goudet O, Kalainathan D, Caillou P, Guyon I, Lopez-Paz D, Sebag M. Learning Functional Causal Models with Generative Neural Networks. In: Escalante HJ, Escalera S, Guyon I, et al., eds. Explainable and Interpretable Models in Computer Vision and Machine Learning. Cham: Springer International Publishing, 2018:39-80.

17. Wang Y, Menkovski V, Wang H, Du X, Pechenizkiy M. *Causal Discovery from Incomplete Data: A Deep Learning Approach*, 2020.

18. Zhu S, Ng I, Chen Z. Causal Discovery with Reinforcement Learning. International Conference on Learning Representations 2020

19. Huang X, Zhu F, Holloway L, Haidar A. *Causal Discovery from Incomplete Data using An Encoder and Reinforcement Learning*, 2020.

20. Designing Optimal Dynamic Treatment Regimes: A Causal Reinforcement Learning Approach. ICML 2020; 2020.

21. Effects of an Angiotensin-Converting–Enzyme Inhibitor, Ramipril, on Cardiovascular Events in High-Risk Patients. New England Journal of Medicine 2000;**342**(18):1376-76 doi: 10.1056/NEJM200005043421819.

22. Rubins HB, Robins SJ, Collins D, et al. Gemfibrozil for the Secondary Prevention of Coronary Heart Disease in Men with Low Levels of High-Density Lipoprotein Cholesterol. New England Journal of Medicine 1999;**341**(6):410-18 doi: 10.1056/nejm199908053410604.

23. Effects of Intensive Blood-Pressure Control in Type 2 Diabetes Mellitus. New England Journal of Medicine 2010;**362**(17):1575-85 doi: 10.1056/NEJMoa1001286.

24. Effects of Intensive Glucose Lowering in Type 2 Diabetes. New England Journal of Medicine 2008;**358**(24):2545-59 doi: 10.1056/NEJMoa0802743.

25. Intensive blood-glucose control with sulphonylureas or insulin compared with conventional treatment and risk of complications in patients with type 2 diabetes (UKPDS 33). The Lancet 1998;**352**(9131):837-53 doi: 10.1016/S0140-6736(98)07019-6.

26. Effects of Combination Lipid Therapy in Type 2 Diabetes Mellitus. New England Journal of Medicine 2010;**362**(17):1563-74 doi: 10.1056/NEJMoa1001282.

27. Cardiovascular Effects of Intensive Lifestyle Intervention in Type 2 Diabetes. New England Journal of Medicine 2013;**369**(2):145-54 doi: 10.1056/NEJMoa1212914.

28. Collins R, Armitage J, Parish S, Sleight P, Peto R. MRC/BHF Heart Protection Study of cholesterol lowering with simvastatin in 20 536 high-risk individuals: A randomised placebo-controlled trial. The Lancet 2002;**360**:7-22 doi: 10.1016/S0140-6736(02)09327-3.

29. Scott R, Brien R, Fulcher G, et al. Effects of Fenofibrate Treatment on Cardiovascular Disease Risk in 9,795 Individuals With Type 2 Diabetes and Various Components of the Metabolic Syndrome. Diabetes Care 2009;**32**(3):493 doi: 10.2337/dc08-1543.

30. Intensive Blood Glucose Control and Vascular Outcomes in Patients with Type 2 Diabetes. New England Journal of Medicine 2008;**358**(24):2560-72 doi: 10.1056/NEJMoa0802987.

31. The Diabetes Control and Complications Trial Research G. The Effect of Intensive Treatment of Diabetes on the Development and Progression of Long-term Complications in Insulin-dependent Diabetes Mellitus. RETINA 1994;**14**(3)

32. Hsu C-y. Benazepril was effective and safe for advanced chronic kidney disease without diabetes. ACP Journal Club 2006;**145**(1):19 doi: 10.7326/ACPJC-2006-145-1-019.

33. Brenner BM, Cooper ME, de Zeeuw D, et al. Effects of Losartan on Renal and Cardiovascular Outcomes in Patients with Type 2 Diabetes and Nephropathy. New England Journal of Medicine 2001;**345**(12):861-69 doi: 10.1056/NEJMoa011161.
